# Assessment of Lagrangian Modeling of Particle Motion in a Spiral Microchannel for Inertial Microfluidics

**DOI:** 10.3390/mi9090433

**Published:** 2018-08-27

**Authors:** Reza Rasooli, Barbaros Çetin

**Affiliations:** Microfluidics & Lab-on-a-Chip Research Group, Mechanical Engineering Department, İ.D. Bilkent University, Ankara 06800, Turkey; r.rasooli90@gmail.com

**Keywords:** inertial microfluidics, Lagrangian discrete phase model, inertial lift

## Abstract

Inertial microfluidics is a promising tool for a label-free particle manipulation for microfluidics technology. It can be utilized for particle separation based on size and shape, as well as focusing of particles. Prediction of particles’ trajectories is essential for the design of inertial microfluidic devices. At this point, numerical modeling is an important tool to understand the underlying physics and assess the performance of devices. A Monte Carlo-type computational model based on a Lagrangian discrete phase model is developed to simulate the particle trajectories in a spiral microchannel for inertial microfluidics. The continuous phase (flow field) is solved without the presence of a discrete phase (particles) using COMSOL Multi-physics. Once the flow field is obtained, the trajectory of particles is determined in the post-processing step via the COMSOL-MATLAB interface. To resemble the operation condition of the device, the random inlet position of the particles, many particles are simulated with random initial locations from the inlet of the microchannel. The applicability of different models for the inertial forces is discussed. The computational model is verified with experimental results from the literature. Different cases in a spiral channel with aspect ratios of 2.0 and 9.0 are simulated. The simulation results for the spiral channel with an aspect ratio of 9.0 are compared against the experimental data. The results reveal that despite certain limitations of our model, the current computational model satisfactorily predicts the location and the width of the focusing streams.

## 1. Introduction

Bio-particle manipulation is an essential process for many biological and biomedical applications. With the help of microfluidics, scientists and researchers have proposed many different techniques to manipulate bio-particles inside microchannel networks [1]. Among other techniques, hydrodynamic-based techniques such as deterministic lateral displacement, pinch flow fractionation, hyrdophoresis and inertial microfluidics offer a label-free manipulation of bio-particles through the interaction of particles with the flow field within a specially-designed microchannel network. In microfluidic applications, typically flow physics is governed by the Stokes equation due to the small Reynolds number (Re) nature of the flow (Re≪1), which means flow follows the boundaries of the domain. Once Re reaches unity, inertial effects begin to present themselves. From the particle dynamics point of view, particles start to migrate across the streamlines due to the existence of inertial lift force and tend to reach an equilibrium position inside a channel. The inertial migration of particles in a circular tube was first reported by Segre and Silberberg [2,3] back in the 1960s. They observed that randomly-dispersed rigid spherical particles at the inlet of a tube migrate to a distance of 0.6-times the radius of tube from the center after a certain distance downstream. Owing to the laminar nature of the flow, equilibrium positions are deterministic. Further studies have revealed that the location of the equilibrium position depends on the flow rate, particle size, channel geometry, as well as the particle shape, which makes particle separation based on size and shape possible [4].

At a finite Re (O(Re)∼1−100), which is referred to as inertial microfluidics, different channel designs with different geometries have been investigated. Due to the simplicity of the fabrication process, straight channels with square or rectangular cross-sections have been mostly investigated in the literature. In a square straight channel, particles fill four focusing positions at symmetrical axes of the channel [5]. On the other hand, for a rectangular straight channel, particles fill only two equilibrium positions placed on the short axis of the channel [6,7,8]. The inertial lift force is responsible for the migration of particles across the streamlines, and hence focusing of particles. Inertial lift force depends on the flow field, particle size, channel and particle geometry and varies within the channel. It is inversely proportional to the particle size and drops drastically for small particles. Typically, the required length of a straight channel to achieve particle focusing yields a microfluidic device with a large footprint. To overcome this issue, serpentine and spiral channels have attracted great attention. In the case of a spiral channel, the curvature introduced along the channel creates a centrifugal pressure difference in the radial direction of the channel due to the velocity mismatch at the center of the channel and the vicinity of the channel wall. This pressure difference induces two symmetric counter rotating vortices known as secondary or Dean flow in the channel cross-section. Dean flow assists particles to migrate faster in the lateral direction by exerting a drag force known as Dean drag on particles. Spiral micro-channels have been extensively exploited for particle separation and filtration. A spiral micro-channel with a rectangular cross-section (with an aspect ratio (AR) 2.0) was exploited for the separation of two different particle of sizes 1.9μm and 7.2μm at different channel Re [9]. It was observed that larger particles migrate toward the inner wall, while smaller particles move toward the outer wall.

Reaching Re in the range of 1–100 inherently dictates relatively high flow rates, hence high throughput (i.e., a large number of bio-particles processed in unit time) for microfluidic devices. High throughput is a critical aspect for clinical applications, for which a great number of bio-particles needs to be processed. One important application is the separation/isolation of circulating tumor cells (CTCs), which are present at an extremely low concentration in whole blood (0–100 CTCs together with $10^{9}$–$10^{10}$ red blood cells and $10^{6}$–$10^{7}$ white blood cells per mL of whole blood). A quantification of the CTCs in peripheral blood or bone marrow is a potential indicator of success or failure of a medical treatment and monitoring of disease progress [10]. In the last decade, many research groups focused on the application of inertial microfluidics for isolation of CTCs [11,12,13,14,15,16,17,18,19,20,21,22,23,24,25]. For sampling CTCs in whole blood, the recovery rate and purity of the isolation are two important metrics along with high throughput [10]. Although inertial microfluidics is a promising technique in terms of throughput, it may have a low recovery rate and/or purity due to the selectivity only based on size. Even though many studies accomplished isolation of CTCs with inertial microfluidics, all of these proof-of-concept studies used cell lines rather than a clinical sample. Unfortunately, although size overlap between the leukocytes and CTCs may not exist for cell lines (please see the size data for the Michigan Cancer Foundation-7 (MCF-7) breast cancer cell line in [26] and size data for white blood cells (WBC) in [27]), overlap may be significant for clinical data (please see the size data for a breast cancer patient in [27]), which would deteriorate successful isolation of CTCs through inertial microfluidics. Considering these challenges for inertial microfluidics, numerical modeling is a crucial tool to predict and assess the underlying physics and performance of inertial microfluidic devices, which will eventually lead to the optimum design with a high throughput, recovery rate and purity.

Strictly speaking, the prediction of a particle trajectory requires the solution of the flow field with the presence of the particle(s). For low Re applications, owing to the linear nature of the governing equations (i.e., Stokes equation), the boundary element method can be implemented for a rigorous simulation of particle motion inside microchannels [28] even with the presence of an external field [29,30,31]. However, inertial effects, which are the driving mechanism for inertial microfluidics, require the solution of non-linear Navier–Stokes equations. Considering a large length-over-diameter ratio and high flow rates for inertial microfluidics, the simulation of particle motion would demand extremely high computational power. With the implementation of periodic boundary conditions for a short section of a long microchannel and/or for a periodic portion of the microchannel network, simulation of flow field with the presence of particles may become feasible [32] (the method utilized in this study is based on the lattice-Boltzmann method rather than the Navier–Stokes equation); however, this is not an option for channels where the radius of curvature of the channel is continuously changing (i.e., spiral channels). Alternatively, if the particle size is small compared to the channel dimensions and the forces acting on the particle are known a priori, a Lagrangian Discrete Phase Model (LDPM) based on the point particle approach can be utilized. LDPM requires the steady solution of the flow field within a microchannel, and calculates particles’ trajectories at the post-processing step. The key ingredient for the simulation of inertial microfluidics is the inertial forces. Inertial forces depend on the flow field, particle size, channel and particle geometry and varies within the channel. Therefore, finding a general solution for the description of inertial forces is most often cumbersome and even impossible. Some studies have been carried out to attempt to find a mathematical description for net inertial lift force. Using the matched asymptotic expansion method, Asmolov [33] derived an analytical solution for the net inertial lift force acting on a small rigid spherical particle flowing in a 2D Poiseuille flow. Very recently, an asymptotic model has been derived for the inertial lift force acting on rigid spherical particles flowing in a 3D Poiseuille flow in square and rectangular (with an AR 2.0) micro-channels [34,35]. Using the immersed boundary integral method, the motion of a spherical rigid particle in a straight square channel was also studied [36]. The effect of high Re was also studied (Reynolds up to 1000). A generalized formula was also proposed for the inertial lift force on a spherical rigid particle in a straight rectangular channel with different ARs [37]. Direct numerical simulation was implemented, and a fitting-analysis was performed to generalize the formula. Once correlations for inertial forces are obtained, the Lagrangian discrete phase model can be utilized. Lagrangian-based modeling is very fast and suitable for Monte Carlo-type simulations where statistical variation of inlet locations, size and properties of particles can be implemented to assess the performance of the process [38,39]. Lagrangian-based modeling has also been implemented for inertial microfluidics using the correlations available for the inertial forces in the literature [19,20,37,40,41].

### Present Study

A computational model based on LDPM is developed to simulate the particle trajectories in a spiral microchannel for inertial microfluidics. The continuous phase (flow field) is solved without the presence of discrete phase (particles) using COMSOL Multi-physics (version 5.0, COMSOL Inc., Stockholm, Sweden). Once the flow field is obtained, the trajectory of particles is determined in the post-processing step via the COMSOL-MATLAB interface. In the post-processing step, a Monte Carlo-type Lagrangian-based modeling is utilized through which Newton’s second law and force balance integration for the discrete phase are implemented to model the particle motion. To resemble the operation condition of the device, many particles are simulated with random initial locations from the inlet of the microchannel. The computational model is verified with experimental results from the literature [9]. Following the verification, the computational model is implemented for particle motion in a spiral channel with different flow rates and with ARs 2.0 and 9.0. Experimental investigations are also conducted for the high AR spiral channel to assess the success of LDPM. Different models are assessed for the implementation of the inertial forces. Hood’s solution, which is derived for straight channels, is implemented in the model for the simulation of spiral channels.

## 2. Computational Model

### 2.1. Generalized Model for Particle Tracking

According to Newton’s second law, the force balance can be written as:

(1) $$\sum F=M_{p}a_{p}$$

where $M_{p}$ is the mass of the particle, F is the equivalent force acting on the particle and $a_{p}$ is the particle acceleration. For curvilinear channels like spiral channels, the cylindrical coordinate system is appropriate. The forces acting on the particle for an inertial microfluidics application can be written in cylindrical coordinates as follows:

(2) $$a_{p}=\left[\begin{array}{c} {\dot{u}}_{p,r}-\frac{u_{p,\theta }^{2}}{r}\\ {\dot{u}}_{p,\theta }+\frac{u_{p,r}u_{p,\theta }}{r}\\ {\dot{u}}_{p,z} \end{array}\right]=\frac{1}{M_{p}}F_{D}+\frac{1}{M_{p}}F_{L}+\frac{1}{M_{p}}F_{B}+\frac{1}{2}\frac{\rho_{f}}{\rho_{p}}\left(a_{f}-a_{p}\right)$$

where $F_{D}$ is the drag force, $F_{L}$ is the lift force, $F_{B}$ is the body force, $\rho_{f}$ is the density of the fluid, $\rho_{p}$ is the density of the particle, and $a_{f}$ is the acceleration of the fluid. $u_{p,r}$, $u_{p,\theta }$ and $u_{p,z}$ are the velocity of the particle in *r*, θ and *z* directions, respectively. A dot over the variable indicates time derivative. The first and second terms are accelerations due to the drag force and inertial lift force. The third term is acceleration due to the body force, which is the buoyancy force in the absence of external fields such as electric, magnetic, etc. Since the density of polymeric microparticles and bioparticles is close to that of buffer solutions in microfluidic applications, the buoyancy term can be neglected. The last term is acceleration due to virtual mass (added mass) force, which is due to the difference in the acceleration of particles and fluid elements surrounding the particles. Assuming the densities of the fluid and particles are the same, and neglecting buoyancy force, the formulation can be rearranged as:

(3) $$\frac{du_{p}}{dt}=\left[\begin{array}{c} {\dot{u}}_{p,r}\\ {\dot{u}}_{p,\theta }\\ {\dot{u}}_{p,z} \end{array}\right]=G=\frac{2}{3M_{p}}\left(F_{D}+F_{L}+\frac{M_{p}}{2}\frac{du_{f}}{dt}\right)+\left[\begin{array}{c} \frac{u_{p,\theta }^{2}}{r}\\ -\frac{u_{p,r}u_{p,\theta }}{r}\\ 0 \end{array}\right]$$

where $u_{p}$ is the velocity of the particle, $u_{f}$ is the velocity of the fluid.

Equation (3) is an initial value problem for which a temporal integration technique needs to be implemented to solve the equation. An implicit integration can be implemented as:

(4) $${u_{p}}^{k+1}={u_{p}}^{k}+\frac{1}{2}\left(G^{k}+G^{k+1}\right)\Delta t$$

where the superscripts (k+1) and (*k*) denote the values at time t+Δt and *t*, respectively. Once the velocity of the particle is known, the new position of the particle can be obtained as:

(5) $${x_{p}}^{k+1}={\left[\begin{array}{c} r\\ \theta \\ z \end{array}\right]}^{k+1}={x_{p}}^{k}+\frac{1}{2}\left\{\begin{array}{c} {\left[\begin{array}{c} \dot{r}\\ \dot{\theta }\\ \dot{z} \end{array}\right]}^{k}+{\left[\begin{array}{c} \dot{r}\\ \dot{\theta }\\ \dot{z} \end{array}\right]}^{k+1} \end{array}\right\}\;\Delta t\;\mathrm{where}\;\left[\begin{array}{c} \dot{r}\\ \dot{\theta }\\ \dot{z} \end{array}\right]=\left[\begin{array}{c} {\dot{u}}_{p,r}\\ \frac{{\dot{u}}_{p,\theta }}{r}\\ {\dot{u}}_{p,z} \end{array}\right]$$

where $x_{p}$ denotes the particle location.

### 2.2. Drag Force

Due to the relative motion of an object in a fluid medium, a resistive force acts on the object in the opposite direction of the motion, known as drag force. Due to the strong dependency of drag force on object properties, it is somehow impossible to propose a general model for drag force. For low Re, the drag force on a spherical particles is given by Stokes’ law:

(6) $$F_{D}=6\pi \mu R_{p}\left(u_{f}-u_{p}\right)$$

where μ is the dynamic viscosity of fluid medium and $R_{p}$ is the particle radius. Stokes’ law is applicable for small Re (≲1) and for a particle in an infinite medium. As Re increases, some corrections need to be included. Typically, these kinds of correlations require a drag coefficient, which is a nonlinear function of Re [42]. Since the time integration in this study requires the value at the latter time step, Stokes’ law is implemented in our study. Regarding the lateral migration velocity of particles, Stokes’ law is acceptable due to the relatively low velocities in the lateral direction. Stokes drag in the axial direction of straight channel is questionable due to high flow rate. To justify this issue, we assume that the particle is released at the inlet with the same velocity as the fluid. By this assumption, the relative velocity of particles with respect to the fluid element in the axial direction becomes relatively small and makes the exploitation of Stokes drag for the axial direction acceptable.

### 2.3. Inertial Lift Model

**Figure 1:**
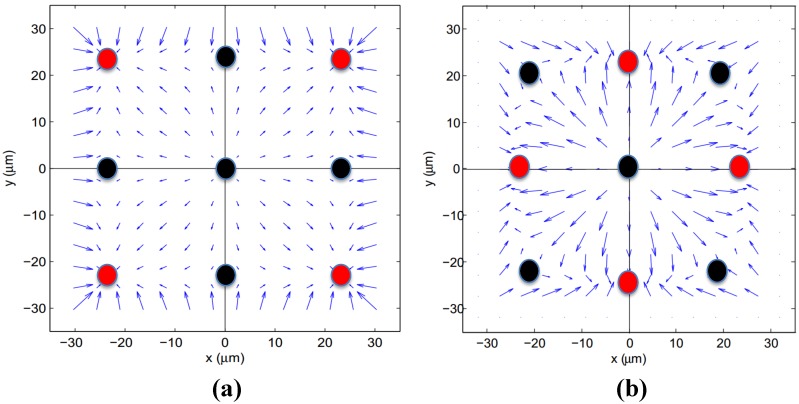
Inertial force field together with stable and unstable equilibrium points in a square channel: (**a**) Asmolov’s study; (**b**) Hood’s study [34] (red points: stable equilibrium; black points: unstable equilibrium).

The inertial lift force field is an phenomena that can be observed in finite Re fluidic systems. Obtaining a solution for the inertial lift force in a desired channel is a cumbersome issue due to the dependency of inertial lift force on many parameters such as channel geometry, channel Re, particle size and properties. Some studies have been carried out to obtain a solution for inertial lift force in simple and basic channel geometries. Unlike drag force, inertial lift force is strongly dependent on the velocity profile and channel geometry. For a rigid spherical particle flowing in a plane Poiseuille flow, Asmolov [33] derived an analytical solution for the inertial lift force based on matched asymptotic expansion:

(7) $$f_{L}=\frac{F_{L}D_{h}^{2}}{\rho U^{2}a^{4}}$$

where $f_{L}$ is the lift force coefficient, $F_{L}$ is the lift force acting on the particle, $D_{h}$ is the hydraulic diameter of the channel, ρ is the density of fluid, *U* is the maximum velocity in the undisturbed velocity profile and *a* is the radius of the particle. The inertial lift coefficient is a function of channel Re and the lateral position of the particle. In this case, the inertial lift force is normal to the walls. The inertial lift force has several components [6]: (i) rotation-induced lift force, (ii) slip-shear-induced lift force, (iii) shear gradient lift force and (iv) wall-induced lift force. Shear gradient-induced and wall-induced lift force are two dominant inertial forces acting on a particle in a plane Poiseuille flow. Shear gradient-induced lift force pushes the particles away from the centerline of the channel due to the curvature of the velocity profile. On the other hand, wall-induced lift force repels the particles away from the channel walls. In a plane Poiseuille flow, the balance of the two aforementioned forces determines the focusing or equilibrium positions of particles in a parallel plate channel. $f_{L}$ indicates the net inertial lift coefficient, which is equal to zero in equilibrium positions. The inertial lift force is higher for larger particles, which results in a faster focusing process. On the other hand, the inertial lift force drastically reduces for small particles and causes particles to reach to their equilibrium positions much more slowly. Re is also a key factor in the determination of equilibrium positions. It was observed that for high Re, equilibrium positions shift towards the channel walls. The solution derived by Asmolov [33] for the inertial lift in plane Poiseuille flow shows a size independent equilibrium position. In other words, this solution predicts the same focusing positions for different sizes of particles. In the case of a square channel, the Asmolov solution can be extended for the determination of the inertial lift force. For this purpose, two different inertial lift components need to be defined. Horizontal and vertical walls induce two inertial lift forces. The resultant inertial lift force field using the Asmolov solution is depicted in Figure 1a. Blue arrays show the direction and also the magnitude of inertial lift force on each point. All the circles included in the figure show the positions where the inertial lift force is zero. Red and black circles indicate stable and unstable equilibrium positions in the channel, respectively. The stability of equilibrium positions can be readily understood from the inertial lift force arrays at the vicinity of the points. For the red circles, which indicate stable equilibrium points, inertial lift force arrows are pointing to the points, and consequently, this causes the particles to return to their equilibrium position with any disturbance. However, any deviation of particles from the black points (i.e., unstable equilibrium points) causes particles to diverge from the unstable points.

Despite the fact that velocity and shear gradient are non-zero only in the lateral direction in a plane Poiseuille flow, they are only zero in the axial direction in 3D Poiseuille flow (i.e., in square and rectangular channels) since the flow is bounded by four solid walls. Although, shear gradient-induced and wall-induced inertial lift forces are the only two dominant forces in a plane Poiseuille flow, Saffman inertial lift force significantly comes into picture for rectangular channels and alters the focusing mechanism in rectangular channels. Moreover, similar to square channels for a rectangular cross-section channel, four equilibrium points near the channel cross-section corners are predicted using Asmolov inertial lift. Unlike these predictions, experiments conducted in the literature show different focusing positions for particles in square and rectangular channels. Different experimental studies carried out for straight rectangular channels [6,7,8] show two equilibrium positions on the short axis of the channel. Although the Asmolov solution has been used in many studies in the literature [19,20,40,41], following these discussions, its application for the prediction of equilibrium points for particles in 3D Poiseuille flow in square and rectangular channels is quite questionable.

Recently, Hood et al. [34] investigated inertial lift force acting on a spherical particle in a 3D Poiseuille flow using asymptotic theory. The resultant inertial lift force field acting on a spherical particle from Hood’s solution [34] is also included in Figure 1b. The figure shows that stable points achieved from Hood’s solution are practically the same as unstable points predicted by Asmolov’s study extended to a square channel. An experimental study using a combination of sub-pixel accurate particle tracking and velocimetric reconstruction of the depth dimension was conducted by Hood [35] for a straight rectangular channel with an AR of 2.0, showing good agreement with Hood’s solution. Alternative to Hood’s solution, more recently, the inertial lift force for a single spherical particle in a rectangular cross-section channel with different ARs had been derived using direct numerical simulation [37]. Compared to the asymptotic solution of Hood, the solutions from the direct numerical solution are more general (i.e., less constraints on flow field and particle shape); however, the implementation of these results for different problems requires a look-up table generated as a result of computationally-heavy direct numerical solutions. On the other hand, Hood’s solution can be generated for a given flow rate, channel geometry and particle size in the form of numerical functions. Since there is no available lift force data for spiral channels, Hood’s solution is utilized for the calculation of the lift force for a given volumetric flow rate, channel size and particle size in this study.

### 2.4. Formulation for Particle Tracking

Introducing the discussed drag and lift forces, parameter G in Equation (4) can be written as:

(8) $$G^{k}=\frac{2}{3M_{p}}\left(6\pi \mu_{f}R_{p}\left({u_{f}}^{k}-{u_{p}}^{k}\right)+{F_{L}}^{k}\left(x_{p}\right)+\frac{M_{p}}{2}\frac{{u_{f}}^{k}-{u_{f}}^{k-1}}{\Delta t}\right)+{\left[\begin{array}{c} \frac{u_{p,\theta }^{2}}{r}\\ -\frac{u_{p,r}u_{p,\theta }}{r}\\ 0 \end{array}\right]}^{k}$$

(9) $$G^{k+1}=\frac{2}{3M_{p}}\left(6\pi \mu_{f}R_{p}\left({u_{f}}^{k+1}-{u_{p}}^{k+1}\right)+{F_{L}}^{k+1}\left(x_{p}\right)+\frac{M_{p}}{2}\frac{{u_{f}}^{k+1}-{u_{f}}^{k}}{\Delta t}\right)+{\left[\begin{array}{c} \frac{u_{p,\theta }^{2}}{r}\\ -\frac{u_{p,r}u_{p,\theta }}{r}\\ 0 \end{array}\right]}^{k+1}$$

Note that the last terms in the parenthesis are discretized using backward and upward integration, respectively. Integration of Equation (4) together with Equations (8) and (9) requires the position and velocity at step (k+1). For the ease of computation, a predictor based on explicit Euler’s method is used for the position:

(10) $${x_{p}}^{k+1}={\left[\begin{array}{c} r\\ \theta \\ z \end{array}\right]}^{k+1}={x_{p}}^{k}+{\left[\begin{array}{c} \dot{r}\\ \dot{\theta }\\ \dot{z} \end{array}\right]}^{k}\Delta t$$

Using the predicted particle location, the velocity of the particle at step (k+1) is obtained from Equation (4). A modified Newton–Raphson method is implemented for the solution of non-linear coupled equations given in Equation (4). Velocity values at step (*k*) are assigned as an initial guess for the non-linear solver. Once the velocity data are available, the location of the particle is corrected using Equation (5).

In curved microchannels due to the existence of a non-zero radius of curvature, a velocity mismatch occurs for fluid elements in the vicinity of the channel walls and central core. This velocity mismatch creates two symmetric circulating secondary flow (Dean) vortices at the cross-section of the channel. The presence of the vortices exerts a drag force known as Dean drag on the particles, which plays a substantial role in size-based particle separation and fast focusing processes. The focusing mechanism in curved microchannels can be justified considering the interaction between inertial lift force and Dean vortices. Lateral migration velocity is solely dependent on the magnitude of inertial lift forces acting on the particles. Therefore, the focusing mechanism of particles in the presence of Dean flow can be explained by the balance of these two forces. The magnitude of these forces is the key point in the determination of the possibility and position of equilibrium points. Once the flow field is determined without the presence of particles, the Dean vortices are present in the flow field. One of the physical mechanism of the inertial lift is the rotational motion, which is inherently absent in the point particle approach; however, the lift force included in our computational model represents the combined effect of the different lift mechanisms; hence, our model inherently includes rotational effects. It should be also noted that although our model somehow accommodates particle-wall interaction through the lift force (the effect of particle-wall interaction on the drag force is not considered), particle-particle interactions are not covered by any means. Therefore, a low particle concentration is an important constraint on our computational model.

## 3. Model Verification

**Figure 2:**
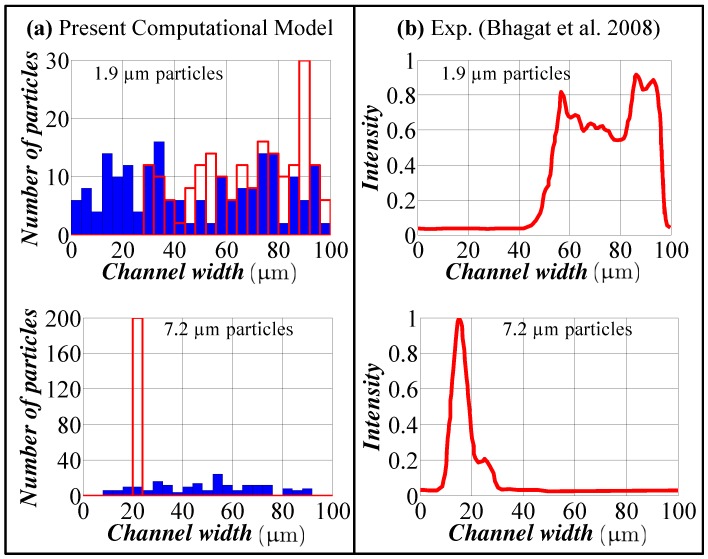
Comparison of the particle distribution for 1.9 μm and 7.2 μm particles (Q = 0.6 mL/h). (**a**) Results from present computational model; (**b**) experimental results (adapted from [9]). Blue bars: particle distribution at the inlet. Red bars: particle distribution at the outlet.

We compare our computational model against the experimental results of Bhagat et al. [9]. As specified in these experiments, a spiral microchannel with a rectangular cross-section (100 μm × 50 μm) and five turns is studied. The starting radius of the spiral is 3.0 mm, and the spacing between two adjacent turns is 200 μm. The flow field is simulated using COMSOL Multi-physics. Due to the symmetry of the problem, only one half of the channel is simulated. Our computational model is implemented in the post-processing step. One hundred particles are released at the inlet with a random distribution (due to the symmetry, this corresponds to 200 released particles). For the channel inlet, laminar inflow with a flow rate of 0.6 mL/h is assigned in accordance with the experimental conditions. The no-slip velocity boundary condition and zero pressure are assigned for the channel walls and channel outlet, respectively. According to the experiment, two different sizes of particle of 1.9 μm and 7.2 μm are considered for the simulation. Figure 2 shows the comparison between simulation and experimental results for 1.9 μm and 7.2 μm particles, respectively. Considering the limitations of our model in conjunction with the use of Stokes’ law and the straight channel results for inertial lift force, our model is able to predict particle behavior and the focusing pattern quite successfully. According to the experimental results, smaller particles tend to focus near the outer wall of the channel, and larger particles tend to focus in the vicinity of the inner channel wall, which is also well predicted by our computational model.

## 4. Computational Results

Following the verification of our computational model, several different cases are simulated to assess the particle focusing of different sizes of particles in spiral microchannels with different ARs (AR = 2.0 and AR = 9.0). Flow fields are obtained solving Navier–Stokes equations using COMSOL Multi-physics. Similar to the simulations for model verification, laminar inflow with the specified flow rate is assigned at the channels’ inlet; zero pressure is assigned at the channels’ outlet; and the no-slip velocity boundary condition is assigned as the boundary conditions for channel walls. The fluid properties are assigned the same as water from the COMSOL library. Free triangular grids with custom properties with a maximum element size of 5.0 μm, minimum element size of 1.0 μm, maximum element growth rate of 1.2, curvature factor of 0.7 and resolution of narrow regions of 0.6 are utilized to generate the mesh for the channel inlet. Using the swept option, a 3D mesh is created along the channel. The flow data are used through the MATLAB-COMSOL LiveLink interface for the particle tracking. The selection of the time step is dependent on the channel flow rates. For low flow rates, a smaller time step needs to be assigned since the particle motion is slower in comparison with higher flow rates.

### 4.1. Spiral Channel with an AR 2.0

**Figure 3:**
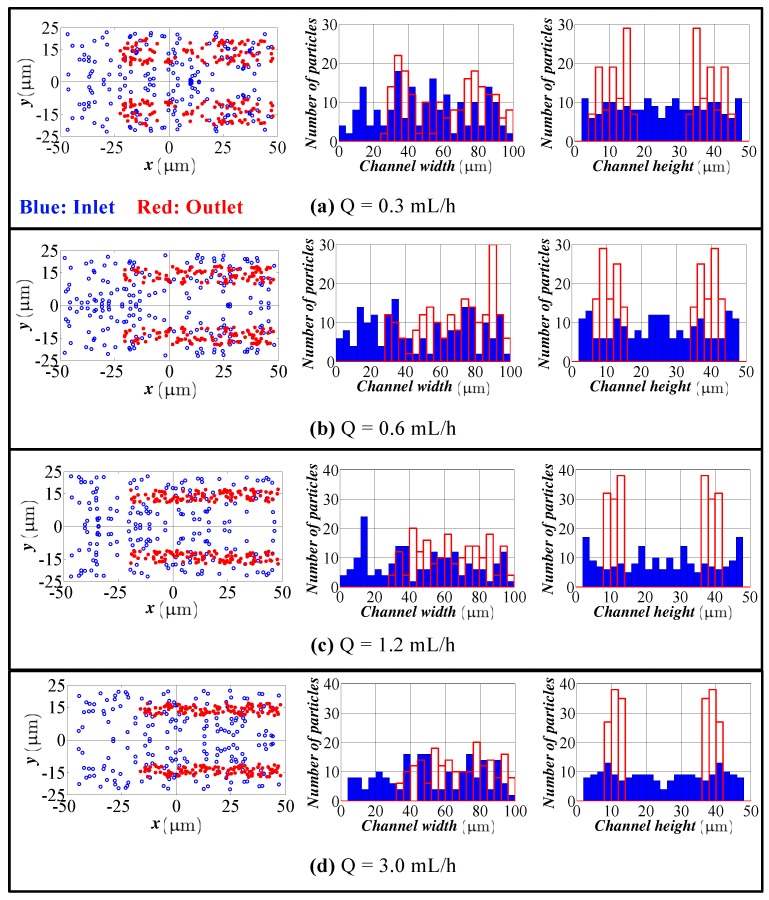
Particle distribution at the inlet and outlet in the height and width directions for different flow rates (spiral channel with an aspect ratio (AR) of 2.0, 1.9 μm particles). Flow rate: (**a**) 0.3 mL/h; (**b**) 0.6 mL/h; (**c**) 1.2 mL/h; (**d**) 3.0 mL/h. Blue bars: particle distribution at the inlet. Red bars: particle distribution at the outlet.

For the channel with an AR 2.0, the same channel used for the model verification is considered. To be consistent with the experimental settings of Bhagat et al. [9], particles with a size of 1.9 and 7.2 μm are simulated. In each simulation, 100 particles were released from the inlet of the channel with a random distribution. The simulation was only performed for the upper half of the spiral channel due to the symmetry, and the results were copied symmetrically for the lower half of the channel. Figure 3 shows the cross-sectional view and particle distributions at the inlet and outlet of the spiral channel for 1.9 μm particles. For the clarity of the figure, the particles are not presented in the actual scale. Different flow rates of 0.3, 0.6, 1.2 and 3.0 mL/h were used, which correspond to channel Re of 1.67, 3.34, 6.67 and 16.67, respectively. The symmetry of the solution can be seen in the figure. The model predicts both focusing in the width and height directions. Particles accumulate towards the outer wall in the width direction. This accumulation creates a wide band. However, the accumulation of particles in the height direction is more pronounced, and actually, particles are accumulated in a relatively tight band. Both the width and height of the focusing region decreases with increasing flow rate. Due to low inertial lift for small particles, Dean drag dominates and causes particles to trap and circulate within Dean vortices; therefore, the focusing region occupies more than half of the channel and closer to the outer wall. One can expect that by increasing the flow rate (which increases the channel Re), the focusing quality can be improved. This expectation is not satisfied in the simulation results. By increasing the channel Re, both inertial lift force and Dean drag are affected and result in rising of the magnitude of both forces simultaneously. The simultaneous increase in the magnitude of both inertial lift force and secondary drag still keeps the ratio of inertial lift and secondary drag very far from unity.

**Figure 4:**
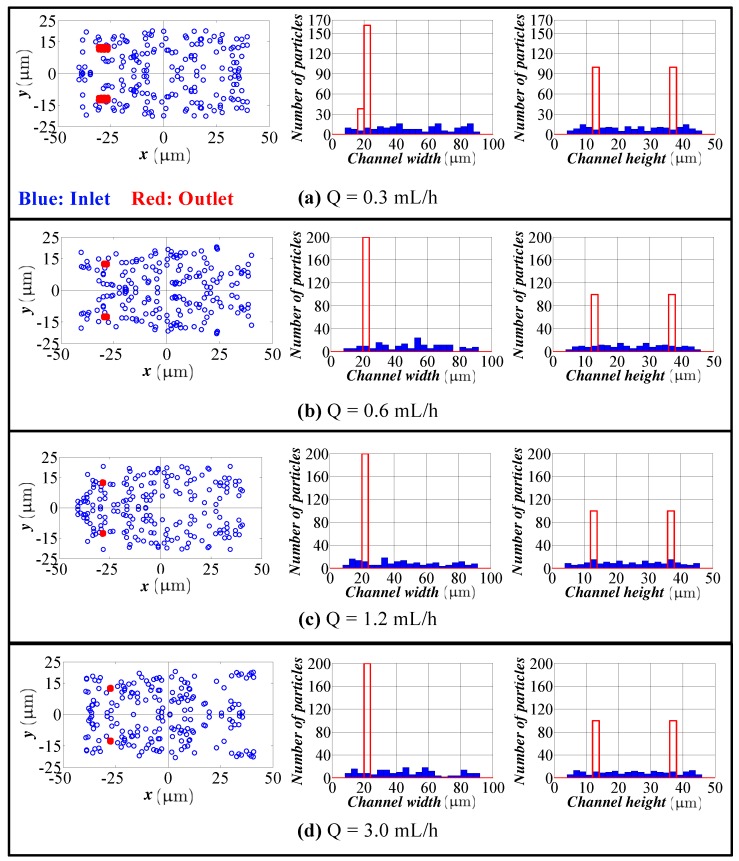
Particle distribution at the inlet and outlet in the height and width directions for different flow rates (spiral channel with an AR 2.0, 7.2 μm particles). Flow rate: (**a**) 0.3 mL/h; (**b**) 0.6 mL/h; (**c**) 1.2 mL/h; (**d**) 3.0 mL/h. Blue bars: particle distribution at the inlet. Red bars: particle distribution at the outlet.

Figure 4 shows the cross-sectional view and the particle distribution at the inlet and outlet of the spiral channel for 7.2 μm particles. In this case, the inertial lift force and Dean drag are virtually in the same order. Now, it is clear that the particles are focused to a point rather than a region even for low flow rates. Focusing quality is improved due to the better balance of the inertial lift and Dean drag. The location of the equilibrium point is independent of the flow rate. In addition, high channel Re results in high velocity shear gradient magnitude in the height direction, which leads to particle focusing also in the height direction.

### 4.2. Spiral Channel with an AR 9.0

A shallow spiral channel with an AR of 9.0 is also simulated to assess the focusing performance. The channel is a spiral with five turns. The starting radius of the spiral is 3.0 mm. The channel has a width of 600 μm and height of 70 μm. The spacing between two adjacent turns is 400 μm. Three different particle sizes with different flow rates are considered for the simulations: Particles with a size of 2.0 μm and 10 μm with flow rates of 40 mL/h, 80 mL/h and 120 mL/h, which correspond to channel Re of 49, 99, 149, respectively; and a particle of a size of 20 with flow rates of 60 mL/h, 80 mL/h and 100 mL/h, which correspond to channel Re of 74, 99, 124, respectively. One hundred particles are released from the inlet of the channel with a random distribution. The channel outlet is placed at the end of the fifth turn of the spiral.

**Figure 5:**
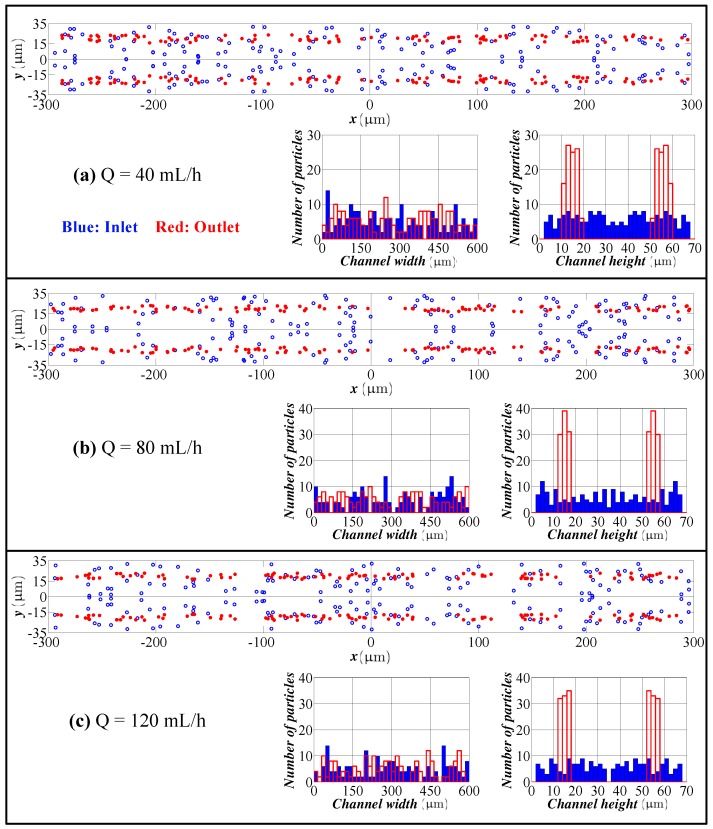
Particle distribution at the inlet and outlet in the height and width directions for different flow rates (spiral channel with an AR 9.0, 2.0 μm particles). Flow rate: (**a**) 40 mL/h; (**b**) 80 mL/h; (**c**) 120 mL/h. Blue bars: particle distribution at the inlet. Red bars: particle distribution at the outlet.

Figure 5 illustrates the cross-sectional view and distribution of particles at the inlet and outlet of the channel for 2.0 μm particles. For small particles, inertial lift force drops substantially, and this issue violates the balance of inertial lift and Dean drag. Due to the high channel hydraulic diameter and small particle size, the balance of inertial lift and Dean drag occur in any region of the channel. Therefore, the particles are trapped in the Dean vortices and circulate within the cross-section. Since a much higher velocity shear gradient exists in the height of the channel and the dominant inertial forces in the height direction are shear gradient-induced and wall-induced lift forces, which are strongly dependent on the velocity shear gradient, particles focus in their equilibrium positions in the height direction. In other words, 2.0 μm particles firstly focus in the height of the channel due to the high velocity shear gradient. Second, they start focusing in the width; however, due to the weaker inertial lift than the Dean drag, they trap in the Dean vortices and circulate at their equilibrium position in the height of the channel. As can be observed from these figures, focusing of 2.0 μm particles has not been achieved in this channel design.

**Figure 6:**
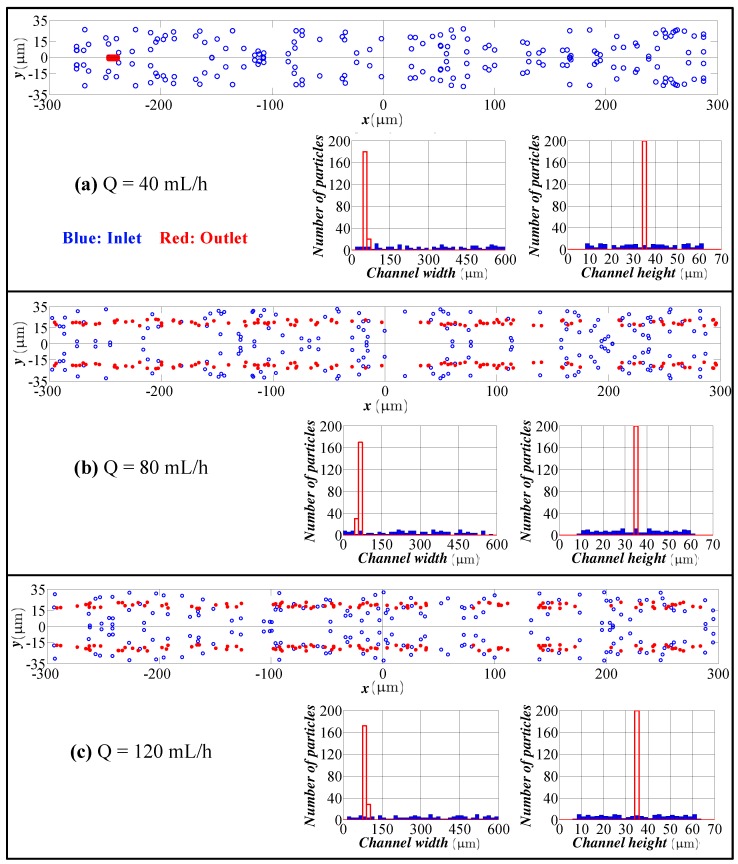
Particle distribution at the inlet and outlet in the height and width directions for different flow rates (spiral channel with an AR 9.0, 10 μm particles). Flow rate: (**a**) 40 mL/h; (**b**) 80 mL/h; (**c**) 120 mL/h. Blue bars: particle distribution at the inlet. Red bars: particle distribution at the outlet.

Figure 6 shows the cross-sectional view and distribution of particles at the inlet and outlet of the channel for 10 μm particles. According to these results, 10 μm particles are focused on the long axis of the channel in the vicinity of the inner wall. For a straight channel with an AR of 2.0, 10 μm particles focus on the long axis of the channel and are symmetrically placed in the vicinity of two vertical walls [43]. Thus, in the spiral channel, particles also focus on the long axis. The existence of secondary flow just relocates the particles’ equilibrium position. Unlike the straight channel in which there are two symmetric equilibrium positions, the spiral channel has only one equilibrium position, which can be attributed to the existence of secondary flow.

**Figure 7:**
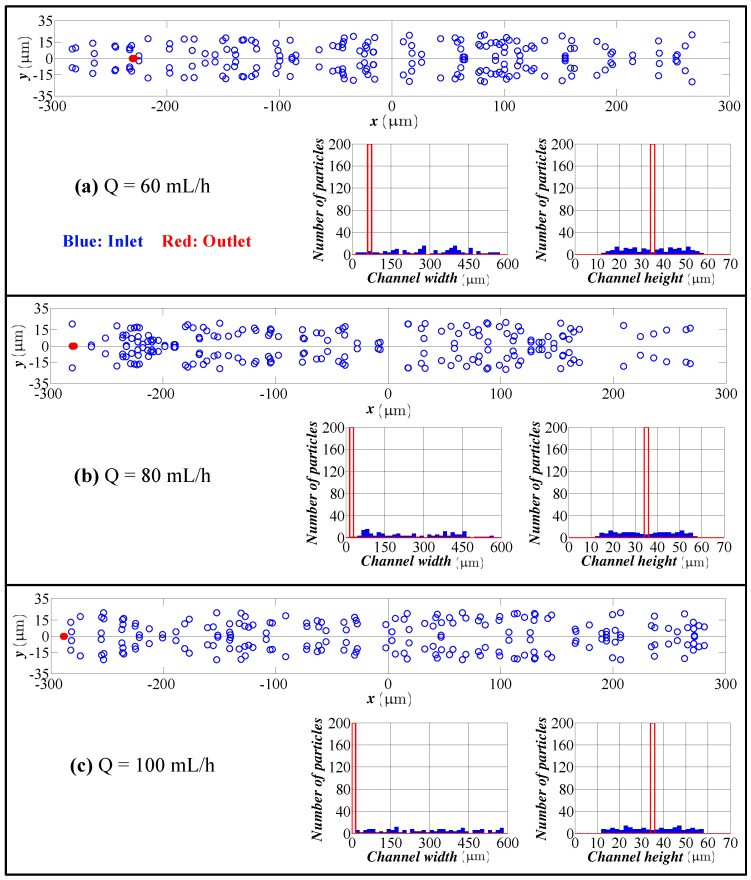
Particle distribution at the inlet and outlet in the height and width directions for different flow rates (spiral channel with an AR 9.0, 20 μm particles). Flow rate: (**a**) 60 mL/h; (**b**) 80 mL/h; (**c**) 100 mL/h. Blue bars: particle distribution at the inlet. Red bars: particle distribution at the outlet.

Figure 7 shows the cross-sectional view and distribution of particles at the inlet and outlet of the channel for 20 μm particles. In this case, the focusing is very clear, and the location of the equilibrium position shifts to the inner wall as flow rate increases.

## 5. Experimentation

### 5.1. Experimental Setup and Procedure

**Figure 8:**
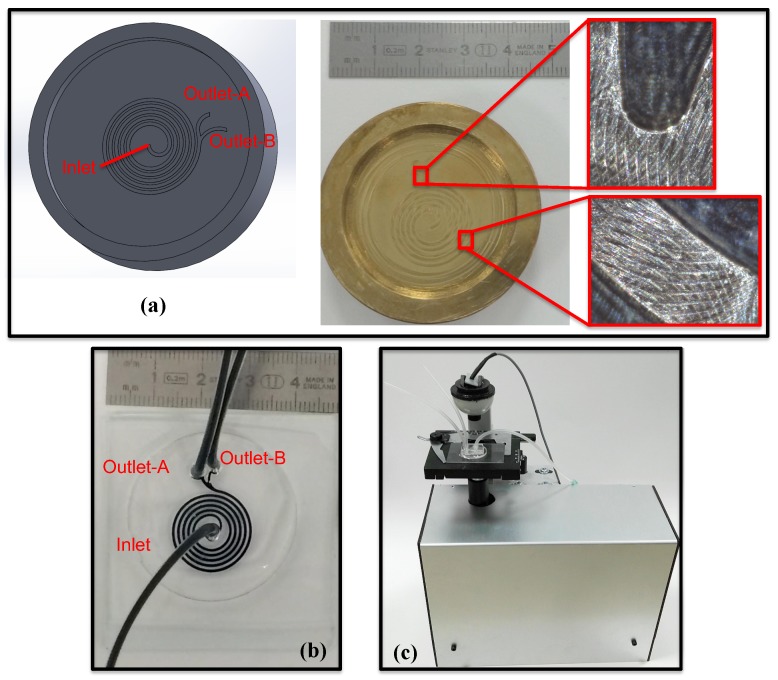
(**a**) Mold of the spiral channel, (**b**) fabricated microfluidic device and (**c**) custom-made epi-fluorescence microscope.

To assess the performance of the computational model, a spiral channel with an AR 9.0 was fabricated. The microfluidic device was fabricated using PDMS molding with a brass mold [39] followed by a plasma bonding on a glass slide. The fabrication of the mold was performed by a 3-axis micro-machining center (PROINO Z3X Micro Maker, Mikro Protez Ltd. Şti., Ankara, Turkey) with an accuracy of 5 μm. Prior to plasma bonding, the peeled PDMS and microscope glass were carefully washed by isopropanol alcohol (IPA) and de-ionized (DI) water to guarantee purified surfaces free of dust and contaminant. Finally, places for the inlet and outlet were punched, and tubings for the inlet and outlet were inserted. To prevent the chip from any leakage due to high pressure at the inlet, a layer of PDMS was poured and baked on top of the chip. To perform the experiments, a solution of micro-particles and DI water with the desired concentration that ensures a low number of particle concentration in the sample solution was prepared. The sample solution was pumped into the micro-channel using a syringe pump with desired flow rates. In order to visualize the particle trajectories and final equilibrium positions, fluorescent latex particles with different sizes together with a custom-made epi-fluorescence microscope were used for small particles of sizes of 10 and 2.0 μm. For 20 μm particles, non-fluorescent particles were also used with a lightening from the top. Different flow rates were assigned at the inlet of channel to investigate the role of Re on the focusing mechanism of particles with different sizes. Figure 8 presents the mold of the spiral channel, fabricated microfluidic device and custom-made epi-fluorescence microscope.

### 5.2. Experimental Results

**Figure 9:**
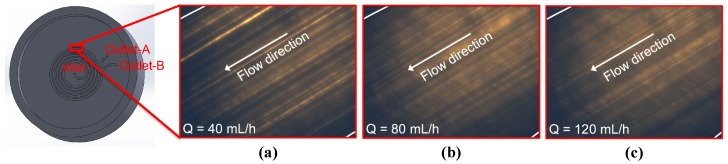
Experimental results for different flow rates (spiral channel with an AR 9.0, 2.0 μm particles). Flow rate: (**a**) 40 mL/h; (**b**) 80 mL/h; (**c**) 120 mL/h.

Figure 9 shows the result of the experiments for 2.0 μm particles. The experiments were conducted for four different flow rates starting from low. Since the microscope focuses on a plane, as predicted by the simulation results, 2.0 μm particles do not focus in the width direction. Simulations predict focusing in the height direction. However, the *z*-direction resolution of our microscope was not small enough to scan the height direction.

**Figure 10:**
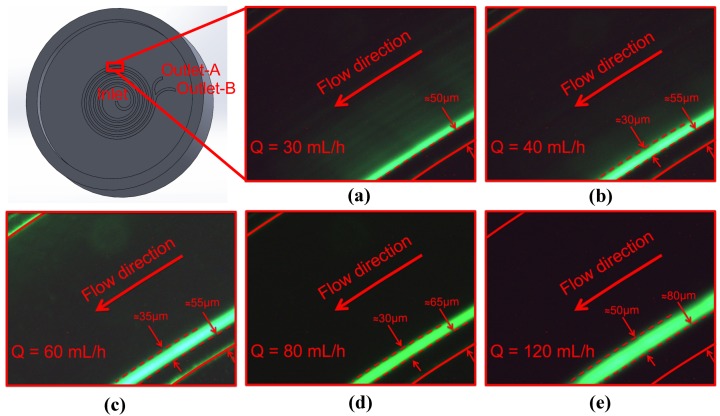
Experimental results for different flow rates (spiral channel with an AR 9.0, 10 μm particles). Flow rate: (**a**) 30 mL/h; (**b**) 40 mL/h; (**c**) 60 mL/h; (**d**) 80 mL/h; (**2**) 120 mL/h.

Figure 10 shows the experiment results for 10 μm particles. The experiments were conducted for a wide range of flow rates and consequently channel Re starting with a flow rate of 30 mL/h. The accumulation of particles starts at the vicinity of the inner wall at the lowest flow rate, but the width of the focusing stream is quite large. Some green shadows can be observed even at the locations close to the center of the channel. For the higher flow rates such as 40 mL/h, 60 mL/h and 80 mL/h, the focusing of particles is fully achieved, and the focusing regions are very clear. This observation shows that for these specific flow rates, the balance of inertial lift and Dean drag is satisfied in a relatively tight region. The quality of focusing is very high for these flow rates since the width of the focusing region is approximately three-times the particle size. For these tight focusing cases, the location of the focused stream is 55 μm–80 μm away from the inner wall. Experiments for 20 μm particles were also conducted; however, the microscope images are excluded so as not to lengthen the manuscript (the microscope images can be found elsewhere [43]).

**Figure 11:**
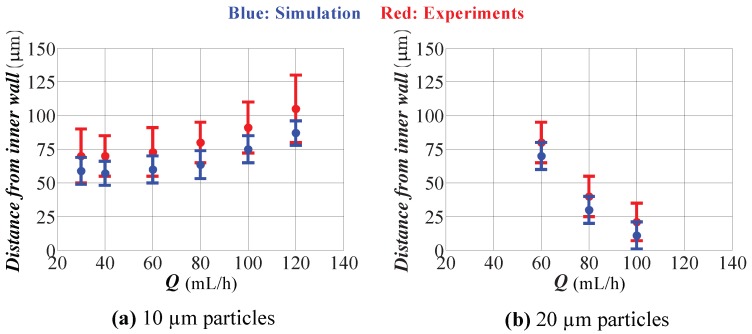
Comparison of the simulations with the experimental results (AR = 9.0). (**a**) 10 μm particles; (**b**) 20 μm particles

The comparison between simulation and experimental results is presented in Figure 11. Experimental data for 10 μm and 20 μm particles are included. The dots represent the center of the focusing region, and the error bars indicate the width of the focusing region. As seen from the figure, simulations predict a closer location to the inner wall than that of the experiments. Moreover, simulation results predict a narrower focusing region than the experiments. This conclusion is valid for all flow rates and for both particles. Considering the limitations of our computational model (use of Stokes drag and inertial lift derived for a straight channel), the comparison can be concluded as satisfactory. The center location of the focusing region is underestimated at most by 15 μm. The simulations predict the width of the focusing region as about 25 μm for all flow rates and for both particles. However, in the experiments, the width of the focusing region increased for 10 μm particles with the flow rate (maximum being 50 μm), and it was approximately 50 μm for 20 μm particles for all flow rates. The discrepancy between the simulations and experiments is really small compared to the width of the channel, which is 600 μm. The location of the focused stream is critical especially for the separation applications. With these results, it is pretty clear that separation of 10 and 20 μm particles is not effective for flow rates smaller than 80 mL/h, and a very efficient separation can be achieved at a flow rate of 100 mL/h.

## 6. Concluding Remarks and Outlook

Inertial microfluidics is a high throughput hydrodynamic-based technique for particle separation and particle focusing for biomedical and biological applications. However, for the development of inertial microfluidic devices with better performance, numerical simulation is an important design tool. Prediction of the flow of particles inside microchannels is crucial. An efficient LDPM-based numerical modeling is presented for the simulation of particle trajectory inside curvilinear microchannels for inertial microfluidics. Our computational model is verified with experimental data of a square spiral channel from the literature and used to assess the focusing performance of square and shallow spiral channels for different particle sizes. The results for the shallow channel are also compared with the experimental observations. Here, are some important aspects and the limitations of the current computational model:

- Since the model is based on Lagrangian modeling, once the flow field is obtained and correlation and/or numerical data are available for the drag and lift forces, simulations of many particles can be performed quite quickly and easily, which makes Monte Carlo-type simulations possible. With Monte Carlo-type simulations, a distribution of particles is obtained that gives realistic predictions for the actual performance.
- In this study, only the inlet location of the particles is varied. If there exists a significant size variation of the particles like in the case of cells, the size variation of the particles can be also included. For a given variation, the computational model can predict a variation of the particles’ location at the exit. Either for focusing or separation applications, the location of the particles at the exit of the microchannel is the important parameter for the design of the outlet section of a microfluidic device.
- The model predicts the position of the particles in the width direction, as well as in the height direction. Although visual inspection in the width direction is straight forward in the experimentation, the data for the height direction is not straight forward and requires a microscope with automated scanning in the height direction with an appropriate image processing software. Therefore, the data for the height direction through a numerical simulation are quite valuable.
- Predictions of the model for the location of the focused particles do not perfectly match with the experiments. This discrepancy is quite expected since our model uses Stokes’ law for the drag calculation, which is valid for a spherical particle in an infinite medium, and Hood’s results, which were derived for a spherical particle in a 3D Poiseuille flow in a straight channel. The model consistently underestimates the width of the focusing region and the location of the focusing region to the inner wall of the spiral channel for different flow rates. Keeping in mind this issue, one can extrapolate the numerical data for a better prediction of the actual operation.
- One important limitation of our computational model is the neglecting of particle-particle interactions. In microfluidics, typically, the number concentration of particles is low to avoid any blockage of the microchannel. Since flow rates are high for inertial microfluidics, blockage is not an issue; therefore the number concentration can be pushed to higher values which may violate the low concentration assumption in our model. The inclusion of particle-particle interactions requires simulations with the presence of the particles, which is a very challenging problem even with today’s computers.
- The current model can be extended for particles with different shapes other than spheres and/or channels with different cross-sectional geometries (one important geometry for inertial microfluidics is a tapered geometry in the literature). However, drag and inertial force correlations are required to employ LPDM for different particles and/or channel geometries. At this point, implementation of direct numerical simulation may be a viable option for the calculation of drag and lift force as proposed by [37].
- Although our model is implemented for a spiral channel, the current model is applicable for the assessment of a general curvilinear channel (e.g., serpentine microchannel).
